# Physical Activity and Its Potential Determinants in Obese Children and Adolescents under Specialist Outpatient Care—A Pilot Cross-Sectional Study

**DOI:** 10.3390/healthcare12020260

**Published:** 2024-01-19

**Authors:** Anna Kawalec, Renata Mozrzymas, Agata Domżol, Agnieszka Zachurzok, Maria Szczepańska, Anna Noczyńska, Danuta Zwolińska

**Affiliations:** 1 Department and Clinic of Pediatric Nephrology, Wroclaw Medical University, Borowska Street 213, 50-556 Wroclaw, Poland; 2 Research and Development Center, Regional Specialist Hospital, Kamieńskiego Street 73a, 51-124 Wroclaw, Poland; 3 Department of Pediatrics, Faculty of Medical Sciences in Zabrze, Medical University of Silesia in Katowice, 3 Maja Street 13/15, 41-800 Zabrze, Poland; 4 Department and Clinic of Endocrinology and Diabetology for Children and Adolescents, Wroclaw Medical University, Chałubińskiego 2a Street, 50-368 Wroclaw, Poland

**Keywords:** physical activity, obesity, children, adolescents, healthcare

## Abstract

(1) Background: this study aimed to assess the physical activity of obese pediatric patients under specialized outpatient care and its potential determinants. (2) Methods: A total of 83 subjects aged 7–18 years with simple obesity and their parents were enrolled. Data were collected with the use of physical activity questionnaires (PAQs) for children and adolescents and additional questions concerning selected socio-demographic characteristics. (3) Results: The mean final PAQ score was 2.09 ± 0.69. The most frequently chosen types of physical activity included walking, gymnastics, and jogging or running. We found a weak correlation inversely proportional between the child’s age and mean final PAQ score (r = −0.25; *p* = 0.02). Younger children were more active during lunchtime at school and after school compared to adolescents (*p* = 0.03 and *p* = 0.04). The final PAQ score differed according to the place of residence; the lowest score was obtained by subjects living in cities >100,000 inhabitants (*p* = 0.025). We found a positive correlation between PAQ-Ch score and the father’s physical activity, and between PAQ-A score and the mother’s education. (4) Conclusions: The physical activity of obese pediatric patients is low, particularly in adolescents. It seems that age and place of residence have an impact on the physical activity of obese children and adolescents. The PAQs used in this study are useful in physical activity assessment and identification of time segments during the day in which activity might be improved. However, this requires confirmation in a larger group of pediatric patients.

## 1. Introduction

Excess weight and its health-related consequences have become a serious problem and one of the major public health issues [1]. According to the World Health Organization (WHO) Obesity Report 2022, almost sixty percent of adults and nearly one-third of children are overweight or obese in Europe [2]. Although current data indicate that in economically developed countries, obesity rates have been stabilized both in adult and pediatric populations [3], during the COVID-19 pandemic, the prevalence of childhood and adolescent obesity significantly increased [2].

In Poland, the estimated prevalence of overweight and obesity in children aged 7–9 years old is 32% and 14%, respectively [4]. These rates decrease with age, as 18% of girls and 34% of boys aged 11 years are overweight or obese, while in 15-year-olds, the percentages are lower both in girls and boys (8% and 23%, respectively) [5]. This trend might be partially explained by changes in body composition, insulin sensitivity, and maturation occurring in adolescents, together with behavioral changes affecting diet, physical and sedentary activity, and socio- and psychological aspects [6].

Obesity is a systemic disease which potentially can affect nearly all organs and systems. The consequences of obesity in pediatric patients impact physical, social, and psychological health [7,8]. These include higher risk for cardiovascular diseases, several endocrine and metabolic disturbances, autoimmune diseases, orthopedic problems, and psychological consequences, such as depression, low self-esteem, anxiety, and social isolation [7,8,9].

Although obesity is a multifactorial disease, it predominantly results from a chronic positive energy balance. Physical activity is a modifiable factor of energy expenditure that accounts for about 20–35% of total energy expenditure [10]. Thus, it is one of the core elements in the prevention and treatment of childhood overweight and obesity [11]. According to the WHO guidelines, children and adolescents aged 5–17 years should do at least an average of 60 min per day of moderate-to-vigorous intensity physical activity across the week (mostly aerobic) and at least 3 days per week of vigorous intensity aerobic activities to strengthen their muscles and bones [12,13]. Also, the amount of time spent on sedentary behavior, particularly recreational screen time, should be limited [12,13]. However, the results of the HBSC study revealed that only 15–24% of school-aged teenagers met these recommendations [5]. Noteworthy, Polish guidelines regarding physical activity for obese children are similar to recommendations dedicated to the general pediatric population [14].

The WHO proposes a multidimensional and multisectoral model of care for the prevention and management of overweight and obesity, with primary healthcare as one of the most essential compounds, integrating tasks and actions from all other healthcare levels. In case there is a need for differential diagnosis of obesity types or specialist care for severe obesity or complex obesity with complications and comorbidities, patients are referred from primary to secondary or tertiary care services and then referred back to primary care for follow-up and monitoring [15].

Similarly, the basic role of primary care in the prevention and treatment of pediatric obesity is underlined in Polish recommendations intitled “Childhood Obesity: Position Statement of Polish Society of Pediatrics, Polish Society for Pediatric Obesity, Polish Society of Pediatric Endocrinology and Diabetes, the College of Family Physicians in Poland and Polish Association for Study on Obesity” [14]. Mazur et al. point out that the diagnosis and treatment of obesity remains one of the main problems both in primary and specialist healthcare in Poland. If the diagnosis of obesity has been made, general practitioners and pediatricians in primary healthcare start appropriate treatment, which includes the education of children and their parents on a healthy lifestyle, recommendations on diet and physical activity, education about the consequences of obesity, information about available support methods, and cooperation with other specialists to improve the effectiveness of care [14].

To date, there were no studies in Poland assessing physical activity among obese pediatric patients under specialist outpatient care. In these patients, education and recommendations for lifestyle changes (based on observation and medical interview) were already initiated at the primary healthcare level. Thus, the aim of this study was to assess the physical activity level of pediatric patients under specialized outpatient care because of obesity and to analyze its potential determinants. In addition, we intended to check the possible usefulness of the physical activity questionnaire for children (PAQ-Ch) and physical activity questionnaire for adolescents (PAQ-A) in the assessment of physical activity levels in healthcare centers.

## 2. Materials and Methods

This study was a pilot cross-sectional analysis of selected determinants of physical activity among pediatric patients referred to the specialist center because of simple obesity and their parents. Four outpatient centers providing specialized care for obese pediatric patients participated in this study. Initially, three centers localized in Wroclaw (Lower Silesian voivodeship) were enrolled. To increase the number of patients and their parents, one more center localized in Zabrze (Upper Silesian voivodeship) was invited.

Inclusion criteria were a diagnosis of simple obesity and age 7 to 18 years old. Exclusion criteria included chronic comorbidities and secondary obesity. Participation in the study was voluntary. In total, 122 patients and their parents were invited to participate in the study, of which 83 patients and 164 parents (83 mothers and 81 fathers) completed the survey.

The study was approved by Wroclaw Medical University Bioethics Committee (opinion No. 707/2022, date of approval 6 October 2022).

### 2.1. The Study Group Characteristics

The study group consisted of 83 pediatric patients under specialist ambulatory care because of obesity (43 boys and 40 girls) and their parents. Patients were divided into two subgroups depending on age and the relevant physical activity questionnaire: children who completed PAQ-Ch (age 7 to 13 years) and adolescents who completed PAQ-A (age 14 to 18 years). Key data regarding study group characteristics are presented in Table 1 and Table 2.

### 2.2. Data Collection

Data were collected by a survey (paper-based or electronic) which was distributed by the doctor or dietitian during a follow-up visit. The survey consisted of 3 parts:

1. Part 1: general information (completed by parents or caregivers):
  - Date of visit, child’s birth date, actual measurements of child’s weight and height obtained during the visit, presence of obesity complications (hypertension, hypercholesterolemia, hypothyroidism, or hyperinsulinemia), and pharmacotherapy.
  - Data regarding parental or caregiver education level (higher education, vocational, upper-secondary or medium, or lower-secondary or elementary), working status (working or not working), and actual measurements of parental weight and height.
  - Place of residence (village, small town < 20,000 inhabitants, town 20,000–100,000 inhabitants, or city > 100,000 inhabitants).
  - Distance from home to school (less than 1 km, between 1 and 2 km, or more than 2 km).
  - Data on how to get from home to school (on foot, by bicycle, school bus or public transport, car, or other).
2. Part 2: parental physical activity (completed by mother and father)—short version of the international physical activity questionnaire (IPAQ).
3. Part 3: patient’s physical activity (completed by patient): the physical activity questionnaire for children (PAQ-Ch) or physical activity questionnaire for adolescents (PAQ-A) according to child’s age.

### 2.3. Assessment of Physical Activity

The patient’s activity level was assessed with the use of PAQ-Ch (for children aged 7 to 13 years) or PAQ-A (for adolescents aged 14 to 18 years). Both questionnaires showed a high reliability and internal consistency in measuring general physical activity levels in youth [16,17,18]. Patients’ physical activity level was calculated according to the PAQ instruction (mean of all items used in the physical activity composite score, with each item valued from 1 to 5). A final score of PAQ-Ch or PAQ-A of 1 indicates low physical activity, whereas a score of 5 indicates high physical activity [16].

Parental physical activity level was assessed by the short version of IPAQ adapted for the Polish population [19]. According to the IPAQ scoring, parental physical activity was classified as high, medium, or low.

### 2.4. Anthropometric Measurements

Body weight and height measurements were taken during the visit. Height was measured barefoot in a standing position with the head in the Frankfurt plane using a stadiometer SECA 213 (Seca GMBH, Hamburg, Germany) with 0.1 cm accuracy. Weight was measured in light clothing, without shoes, on an electronic scale SECA 875 (Seca GMBH, Hamburg, Germany) with 0.05 kg accuracy. Three measurements were taken, and the mean value was recorded. The online calculator using the Polish growth charts was used to calculate patient’s BMI and assess BMI percentiles (cut-off for obesity > +2SD according to WHO) [20].

Parental BMI was calculated based on self-reported weight and height and classified according to its value as normal (18.5–24.9 kg/m^2^), overweight (25–29.9 kg/m^2^), or obese (≥30 kg/m^2^). There were no parents with underweight (BMI < 18.5 kg/m^2^) in the study group.

### 2.5. Statistical Analysis

Collected data were inserted into a Microsoft Excell spreadsheet. Statistical analysis was performed using Microsoft 365 Excel and STATISTICA 13 software.

For all quantitative variables, mean value (M), standard deviation (SD), median (median), the first and third quartiles (Q1 and Q3), and volatility range (min and max) were calculated.

A comparison of the prevalence of obesity complications between children and adolescents was conducted with the use of the exact Fisher test.

**Figure 1:**
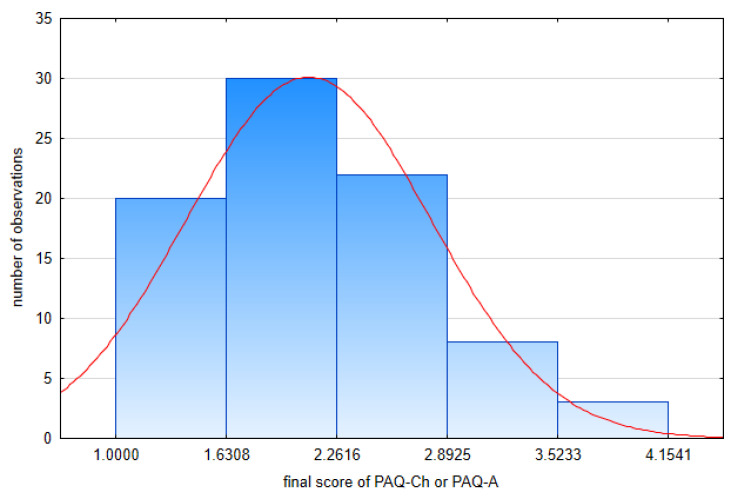
Distribution of final PAQ-Ch and PAQ-A scores.

The normal distribution of each patient’s final PAQ score was checked with the one-sample Kolmogorov–Smirnow test (statistic D 0.1185, critical D (D_crit_) value of 0.1491 for n = 83 and α = 0.05 according to Kolmogorov–Smirnow table). Since D < D_crit_, approximate normality was assumed for further analysis (Figure 1).

The significance of differences in the final PAQ score between girls and boys was checked with Student’s *t*-test. Similarly, Student’s *t*-test was used to check differences between children’s and adolescents’ responses in selected PAQ items, with each item valued from 1 to 5 (activity in the last 7 days during physical education classes, at lunch, right after school, in the evening, and on the last weekend). Differences in the final PAQ score between three or more patient subgroups were tested with ANOVA (subgroups were divided according to place of residence, parental physical activity, or parental education).

The Pearson correlation coefficient (r) was generated to examine the association between the final PAQ score and the patient’s age. To check associations between the final PAQ score and qualitative variables (place of residence, distance from home to school, parental physical activity level, and parental education), Spearman’s correlation coefficients (rho) were generated. The correlations were checked for the whole study group and separately for children and adolescents.

A general regression model was used to identify possible predictors of a patient’s final PAQ score. Due to incomplete parental data, data regarding parental education level, physical activity, and BMI were unified into categories: at least one parent with higher education, at least one parent with moderate or high physical activity level, and at least one obese parent. Also, the mode of commuting to school was unified into two categories: active (on foot or by bicycle) or not active (school bus/public transport or by car). The initial regression model was developed using a set of ten predictors (candidate variables): gender (male = 0, female = 1), age, at least one parent with higher education (no = 0, yes = 1), mother’s working status (not working = 0, working = 1), father’s working status (not working = 0, working = 1), at least one obese parent (no = 0, yes = 1), place of residence (village = 0, small town = 1, town = 2, or city = 3), distance from home to school (0 = below 1 km, 1 = 1–2 km, or 2 = above 2 km), active commuting to school (no = 0, yes = 1), and at least one parent with moderate or high physical activity level (no = 0, yes = 1). The general regression model enabled the inclusion of the quantitative variable (age) and qualitative variables (remaining categories). A series of regression analyses were conducted using a stepwise backward selection method (the least significant predictors were progressively dropped out of the model until only those significant remained).

The significance level was assumed as *p* < 0.05.

## 3. Results

### 3.1. Obesity-Related Complications

The prevalence of obesity-related complications in the whole study group was as follows: hypertension 17.3%, dyslipidemia 7.4%, hypothyroidism 10%, and prediabetes (hyperinsulinism) 9.9% (Table 3). We noted significant differences in the prevalence of hypertension between children and adolescents (2.4% vs. 32.5%, *p* < 0.05). There were no differences in the prevalence of other assessed complications.

### 3.2. Parental Physical Activity

The data characterizing parental physical activity are presented in Table 4.

### 3.3. Patient’s Physical Activity and Its Determinants

The mean final PAQ-Ch and PAQ-A score was 2.09 ± 0.69. Descriptive statistics of the final scoring obtained by pediatric obese patients are presented in Table 5.

**Figure 2:**
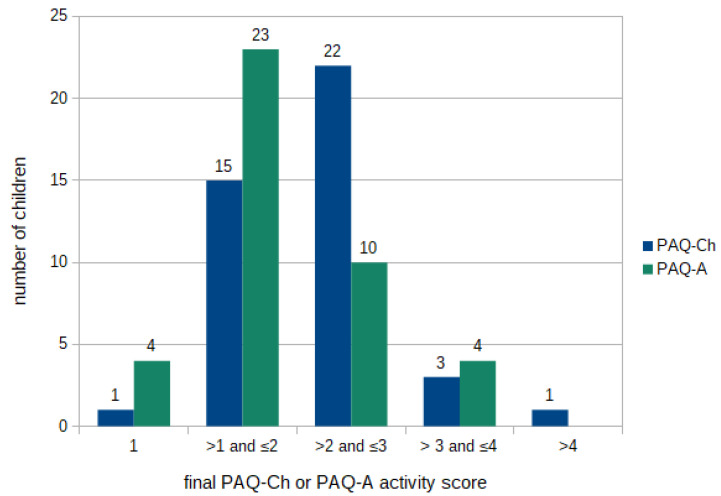
Final PAQ-Ch and PAQ-A scoring.

In total, the score of 1 was obtained by five patients (6.0%), while only one patient obtained a score above 4 (Figure 2).

**Figure 3:**
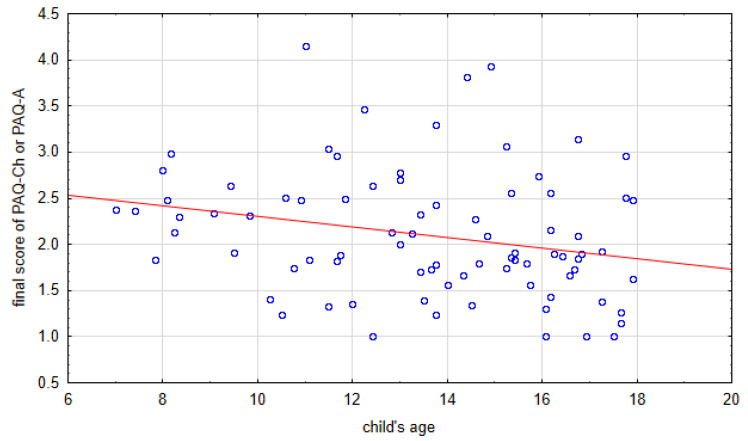
Correlation between child’s age and physical activity level assessed by PAQ-Ch or PAQ-A.

Further analysis showed a statistically significant inversely proportional weak correlation between patient’s age and final scoring of PAQ-Ch or PAQ-A (r = −0.25, *p* = 0.02). Younger children presented higher levels of physical activity and obtained higher PAQ scores than older children (Figure 3).

#### 3.3.1. Correlations between Children’s and Adolescents’ Physical Activity and Selected Parental and Environmental Parameters

The analysis of bivariate correlations between final PAQ score and selected parental and environmental parameters showed no significant associations for the whole study group. The separate analysis according to patient’s age revealed a positive correlation between father’s physical activity and child’s physical activity and a positive correlation between mother’s education and adolescent’s physical activity (Table 6).

#### 3.3.2. Patient’s Physical Activity according to Gender

**Figure 4:**
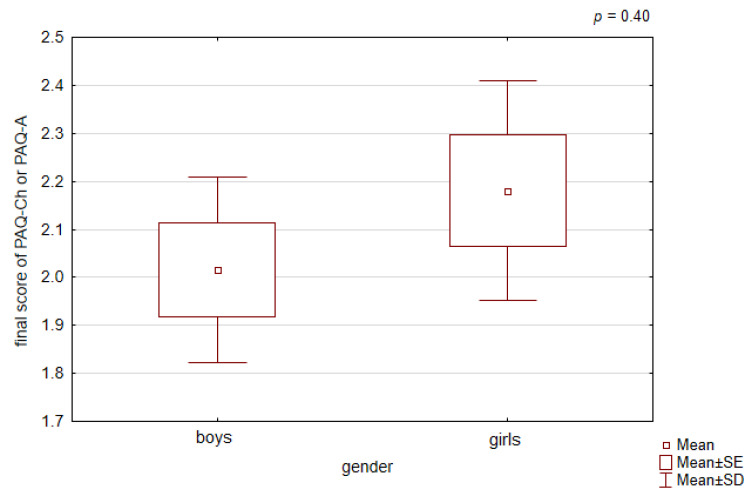
Final PAQ score according to gender.

We found no significant differences in physical activity scores between girls and boys (the mean final PAQ score was 2.18 vs. 2.02; Student’s *t*-test *p* > 0.05, Figure 4).

#### 3.3.3. Children’s and Adolescents’ Physical Activity in Different Time Segments

Younger children were more active during lunchtime at school and after school as compared to adolescents (Student’s *t*-test, *p* = 0.03 and *p* = 0.04, respectively). There were no significant differences concerning evening activity (Student’s *t*-test, *p* = 0.91) nor weekend activity (Student’s *t*-test, *p* = 0.19) between these age groups.

#### 3.3.4. Children’s and Adolescents’ Physical Activity according to the Place of Residence

**Figure 5:**
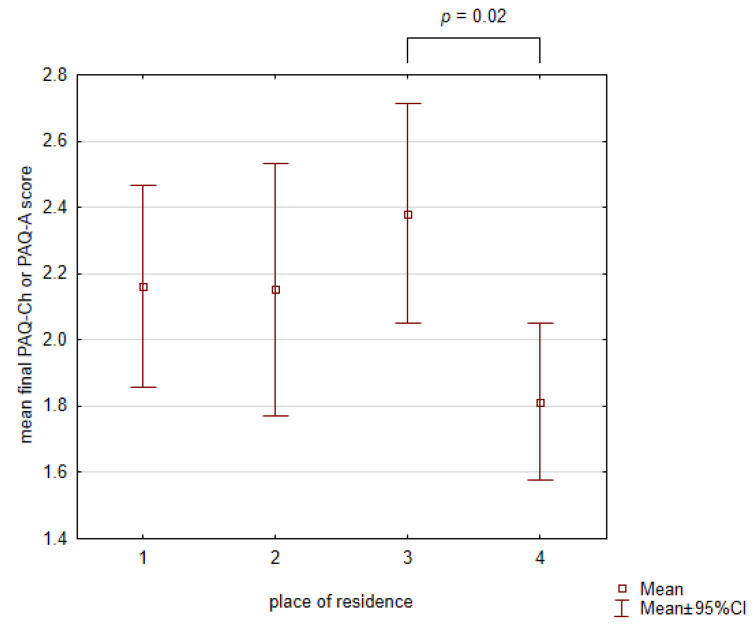
Mean final PAQ score and the place of residence (*X*-axis labels: 1—village, 2—small town < 20,000 inhabitants, 3—town 20,000–100,000 inhabitants, and 4—city > 100,000 inhabitants). CI—confidence interval.

A comparison between the groups according to the place of residence showed differences in physical activity level (Figure 5). The lowest final score of PAQ-Ch or PAQ-A was obtained by subjects living in cities > 100,000 inhabitants and the difference was statistically significant compared to those living in towns with 20,000–100,000 residents (*p* = 0.02; ANOVA and post-hoc Tukey test).

#### 3.3.5. Children’s and Adolescents’ Physical Activity according to Parental Physical Activity and Education

**Figure 6:**
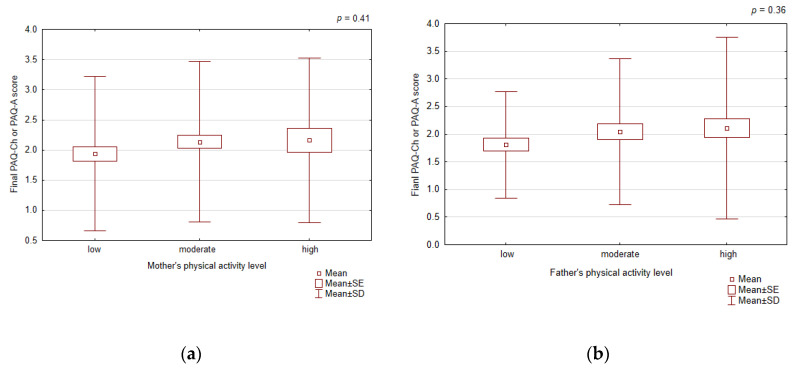
The final PAQ-Ch or PAQ-A score according to mother’s (**a**) and father’s (**b**) physical activity.

The father’s and mother’s physical activity level classified according to IPAQ did not correlate with the child’s physical activity expressed as the final score of PAQ-Ch or PAQ-A (Figure 6).

**Figure 7:**
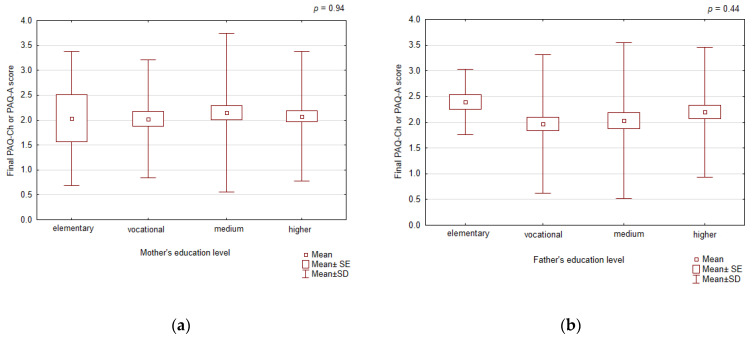
The final PAQ-Ch or PAQ-A scoring according to mother’s (**a**) and father’s (**b**) education.

We found no differences in the final score of PAQ-Ch or PAQ-A according to the mother’s or father’s education (Figure 7).

#### 3.3.6. Children’s and Adolescents’ Physical Activity in Spare Time

**Figure 8:**
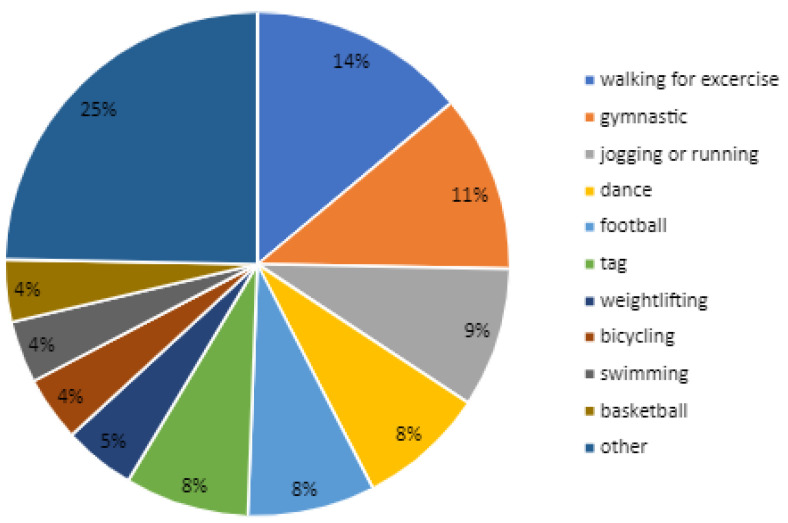
Physical activity during spare time. Category “other” includes other types of physical activity listed in the first PAQ item.

The most frequently chosen types of physical activity during free time among obese pediatric patients were as follows: walking for exercise, gymnastics, jogging or running, dance, football, and tag (Figure 8).

#### 3.3.7. Regression Model—Factors Affecting Children and Adolescent’s Physical Activity

The subsequent regression models were built with a stepwise backward variable selection method (Supplementary Table S1). In the final model, the patient’s age was the only significant variable predicting PAQ score (Table 7).

## 4. Discussion

This study aimed to assess physical activity and its determinants among pediatric obese patients. Our results showed that the physical activity of pediatric patients with obesity under specialist outpatient care is quite low. This observation is supported by the low mean final PAQ score obtained for the entire study group, especially in adolescents. Noteworthy, this is a particular group of patients and their parents that was educated on a healthy lifestyle and was instructed on the recommendations for physical activity at the very beginning of the treatment. The instructions were provided by their primary healthcare worker and repeated during the visit to a specialist center. It should be stressed that our study assessing the physical activity of obese children and adolescents using PAQ is the first in Poland. Nevertheless, Wyszyńska et al., using the same tool to examine the overall physical activity of Polish school children aged 11–13 years, showed higher values of PAQ scores (2.86 ± 0.89) than our study group [21]. These results are not surprising and confirm the usefulness of the PAQ for assessing physical activity.

Our findings are in line with the results of others indicating that obese children present low levels of physical activity [22]. A systematic review by Elmesmari et al., which included 26 studies, reported that the physical activity of obese children and adolescents is below the recommended 60 min of moderate-to-vigorous physical activity per day and is significantly lower than in non-obese peers [23]. The decline in physical activity with age in the general pediatric population has also been well described. In our study, we observed an inversely proportional correlation between age and physical activity. Although adolescence is presumed to be a critical period in which the physical activity level decreases, new data suggest that this process begins around 6–7 years and that there is another drop at the age of 13 years [24,25,26,27].

Interestingly, we observed that periods during the day in which children and adolescents are more active vary according to the age group. Younger children tend to be more active during lunchtime at school and after-school periods, as compared to adolescents. Similar trends were described for the general pediatric population. Brooke et al., comparing the physical activity of 10- and 14-year-olds, reported its decline in all time segments during the day with age, in particular on weekends, out-of-school, and during lunchtime [28]. Similarly, Saint-Maurice et al. indicated that children attending elementary school were more active during lunchtime and after school than those attending middle or high school [29]. This may reflect the impact of school facilities and school environment on the patterns of a child’s activity. According to Lanningham-Foster et al., activity-permissive school environments (which are equipped with activity-related tools and structures and allow more movement) enhance children’s physical activity [30]. Also, the transition from elementary to secondary school significantly impacts a child’s physical activity [31]. The time spent at school and during after-school period hold potential for increasing a child’s physical activity level [32,33]. It is estimated that physical activity during lunchtime at school accumulates approximately 15% of total daily activity and more than half of daily steps taken are attributable to after-school activities [32]. Thus, strategies to increase physical activity at school are a core element of a multidimensional approach not only in the prevention but also in the treatment of childhood overweight and obesity. The identification of time segments during the day in which a child could be more active might help healthcare workers in giving tailored and adequate advice regarding lifestyle modifications for obese children and their parents.

Our analysis of possible factors related to physical activity in obese children and adolescents revealed that the physical activity level differs according to the place of residence. The lowest physical activity was observed in subjects living in a large agglomeration, in cities > 100,000 inhabitants. The urban–rural disparity in the prevalence of overweight and obesity and physical activity level has been extensively investigated; however, the obtained results are inconsistent. Some studies reported that urban children are less active than rural and suburban peers, whereas other research showed no differences in implementations of the recommendations for moderate-to-vigorous physical activity between children living in rural and urban areas [34]. The built environment influences physical activity or inactivity, with significant differences in the physical activity level of residents of the most and least activity-friendly neighborhoods [35]. A systematic review evaluating the built environment’s impact on physical activity and obesity among children and adolescents showed that accessible parks, walking and biking paths, recreational facilities in the nearest neighborhood, and lower residential density might increase children’s engagement in physical activity [36]. Although a built environment did not appear to be a barrier to active living, it is uncommon for obese youth to walk or bike to close locations within 2 km distance [37]. Differences in physical activity among Polish teenagers according to their place of residence have been observed. Those living in urban areas more often declare active lifestyles and spend more hours per week performing sports than those in rural areas [38]. In contrast, the results of “the ABC of Healthy Eating Study” showed the regional differences in activity–inactivity patterns among teenagers, with the most unfavorable and inactive pattern positively associated with urban residence [39]. There are also urban–rural differences in the school environment related to physical activity; for instance, the school location determines the availability of an indoor gym for pupils [40]. Although the regional differences in the prevalence of excess weight among Polish children have been well described [38,39,41,42], there are no studies on the relationship between the place of residence and physical activity in obese pediatric patients.

The results of our correlation studies regarding patient’s physical activity and parental physical activity did not show any relationship. The additional age-specific analysis revealed a positive correlation between the father’s physical activity and the child’s physical activity. However, this correlation was insignificant for adolescents. The literature data on this topic remain inconclusive [43,44]. It is presumed that active parents have more active children, and the impact of parental modeling on child physical activity is context-specific and related to gender [45,46,47]. According to the recent systematic review by Petersen et al., the majority of evaluated research studies show a weak positive association between parental and child physical activity regardless of the child’s age and gender of the parent–child dyads [48]. Similarly, a systematic review by Matos et al. pointed to a correlation between parents’ and children’s physical activity, but with a significant and positive trend toward the same gender dyads (father/son; mother/daughter) [49]. While taking into account obese children and their parents, the evidence from the intervention studies also suggests that parental physical activity influences child physical activity. A study by Foote et al. showed a significant positive relationship between maternal and child physical activity measured in the number of steps took by the mother and their child during the family-based fitness intervention [50].

Considering potential socio-demographic characteristics of the family related to the physical activity of obese pediatric patients, we found a positive correlation between the mother’s education and the adolescent’s physical activity, but this was observed only in this age group. The evidence indicates the strong associations between parental education and employment status and the prevalence of family obesity [51]. The family socio-economic status impacts the physical activity level of the children and adolescents [52,53]. Parental education is connected with their health literacy and combined with financial resources, might influence a child’s physical activity [52,53,54]. This may be due to the parent’s attitude toward health behaviors, affordability, and accessibility to after-school activities, including structured sports activities, as well as the characteristics of the school and neighborhood environment [52,53]. This association was also described among Polish adolescents by Górnicka et al. who showed that the lower socio-economic status of the family, the higher the teenager’s adherence to unfavorable low physical activity patterns [39].

Despite the efforts undertaken by healthcare workers in primary and specialist outpatient care, the results of this study show that the physical activity level of obese pediatric patients is insufficient. Interventions aiming to increase this activity are integral elements of a holistic approach toward long-term lifestyle changes and include the provision of education for children and their parents and/or a structured exercise program [24]. There is no consensus regarding the effectiveness of currently available interventions aiming to increase physical activity in obese children and adolescents [55]. However, it is suggested that the most efficacious actions consist of sessions lasting at least 60 min on three or more days per week for at least 12 weeks [24]. While taking care of obese patients, children and their parents are educated on the role of physical activity in energy balance, the benefits of regular activity in weight reduction, and the management of obesity-related complications. Nevertheless, our study points toward strengthening the efforts and searching for more compelling education methods and providing more tailored and individualized advice. An accurate physical activity level assessment might help to specify the main problems and give directions for possible modifications of everyday activity patterns. The availability of equipment for objective measurements of physical activity is quite limited in primary care and specialist centers. Thus, it seems that the use of standardized questionnaires for physical activity assessment might be of help. Based on our study and observations, the PAQ-Ch and PAQ-A could be proposed as additional support in the child’s physical activity assessment during the patient’s medical history collection and interview. Both questionnaires are already translated and validated for the Polish population and have been successfully used among different groups of children [21,56,57]. Our results show that PAQ-Ch and PAQ-A are helpful in a group of pediatric obese patients, both during the first assessment and follow-up.

It is worth emphasizing that apart from physical activity, the implementation of dietary modifications, psychological support, and behavioral interventions is also essential in obesity management. Among several barriers to physical activity reported in youth with excess weight, a lack of motivation seems to be an important factor [37,58]. Based on our results, we plan to design an educational game on energy balance and physical activity for children and their parents. Of note, precautions must be considered due to specific musculoskeletal conditions in obese youth to secure the patient’s safety.

### Study Limitations

Our study has some limitations. The pilot cross-sectional design does not allow for the conclusion of causality. The present study’s sample size was relatively small. The limited number of participants poses a challenge in comprehending whether discrepancies between our results, particularly the regression model, and the results from other studies can be attributed to this small sample size. The survey that we used assessed only selected factors which might affect patient’s physical activity; for example, there were no questions about dietary habits, sedentary activities, characteristics of a built environment, psychological aspects, and motivation. Like in all voluntary survey-based studies, there is a risk of bias due to non-random study group selection, recall, and self-report bias. In addition, for the paper-based questionnaire, we faced the problem of missing data. Another consideration is subjective assessment of physical activity. Objectively measured physical activity or using mixed methods should be implemented in further research. Although this study used non-objective methods for the assessment of the physical activity level of participants, to ensure better data quality, we decided to apply standardized and adapted for the Polish population questionnaires of PAQ-Ch or PAQ-A and IPAQ. As there was no control group in our study, the general interpretation of high or low activity is limited. Nevertheless, for the patient’s physical activity assessment, we used instructions provided with the PAQs and compared the PAQ score obtained by our patients with the PAQ score obtained by Polish healthy children. Despite the limitations mentioned above, the results of this study enrich the knowledge on this topic and might contribute to better organization of the healthcare provided to obese pediatric patients in primary and specialist centers. Further studies on a larger group of obese patients are required to search for and develop more effective education methods on physical activity to be used during the visit to healthcare centers and strategies to motivate patients to apply beneficial lifestyle changes, with the assessment of their impact in short- and long-term follow-up.

## 5. Conclusions

The physical activity of obese pediatric patients is low, particularly in adolescents. It seems that a child’s age and place of residence have an impact on the physical activity in this group of patients. The PAQs are useful tools for physical activity assessment and the identification of the time during the day in which this activity might be improved. However, this requires confirmation in a larger group of pediatric patients.

## Figures and Tables

**Table 1:** Patients’ characteristics.

	PAQ-Ch	PAQ-A	Whole Group (PAQ-Ch and PAQ-A)
Number of patients	42	41	83
Gender			
Female	19 (45.2%)	21 (51.2%)	40 (48.2%)
Male	23 (54.8%)	20 (48.8%)	43 (51.8%)
Age [years]			
Mean ± SD	11.2 ± 2.1	16.1 ± 1.1	13.6 ± 3.0
Median [Q1; Q3]	11.6 [9.6; 13]	16.2 [15.3; 16.9]	13.75 [11.6; 16.2]
Min–Max	7–13.8	14–17.9	7–17.9
Weight [kg]			
Mean ± SD	79.6 ± 23.5	102.6 ± 20.0	91.4 ± 24.5
Median [Q1; Q3]	76.7 [63.3; 93.5]	99.0 [88.8; 115]	92.0 [75; 106.8]
Min–Max	45–158	60–142	45–158
Height [cm]			
Mean ± SD	153.1 ± 12.7	169.9 ± 8.2	161.7 ± 13.5
Median [Q1; Q3]	155.5 [145.4; 162.6]	170 [163.8; 174.3]	163 [155.3; 171.8]
Min–Max	127–176	150–190	127–190
BMI [kg/m^2^]			
Mean ± SD	33.6 ± 7.0	35.5 ± 6.7	34.6 ± 6.9
Median [Q1; Q3]	32.2 [28.8; 40.3]	33.9 [30.9; 39.4]	33.4 [29.7; 40.3]
Min–Max	23.3–51.0	26.0–53.4	23.3–53.4
BMI percentiles			
Mean ± SD	98.8 ± 1.4	98.9 ± 1.5	98.9 ± 1.4
Median [Q1; Q3]	99.0 [98.0; 99.9]	99.9 [98.8; 99.9]	99.9 [98; 99.9]
Min–Max	95.0–99.9	95.0–99.9	95.0–99.9
Place of residence			
Village	9 (21.4%)	9 (21.9%)	18 (21.7%)
Small town < 20,000 inhabitants	6 (14.3%)	6 (14.6%)	12 (14.5%)
Town 20,000–100,000 inhabitants	11 (26.2%)	12 (29.3%)	23 (27.7%)
City > 100,000 inhabitants	16 (38.1%)	14 (34.2%)	30 (36.1%)
Distance from home to school			
<1 km	16 (38.1%)	11 (26.8%)	27 (32.5%)
1 to 2 km	14 (33.3%)	8 (19.5%)	22 (26.5%)
>2 km	12 (28.6%)	22 (53.7%)	34 (41.0%)
Commuting from home to school			
On foot	20 (48.8%)	14 (35.0%)	34 (42.0%)
By bicycle	2 (4.9%)	1 (2.5%)	3 (3.7%)
School bus/public transport	4 (9.8%)	18 (45.0%)	22 (27.2%)
Car	15 (36.6%)	6 (15.0%)	21 (25.9%)
Other	0	1 (2.5%)	1 (1.2%)
Missing data	1	1	2

**Table 2:** Parents’ demographic data and BMI assessment.

	Mother	Father
Education level		
Higher education	32 (38.5%)	24 (29.6%)
Upper-secondary or medium	33 (39.8%)	23 (28.4%)
Vocational	16 (19.3%)	29 (35.8%)
Lower-secondary or elementary	2 (2.4%)	5 (6.2%)
Missing data	0	2
Working status		
Working	69 (83.1%)	79 (97.5%)
Not working	14 (16.9%)	2 (2.5%)
Missing data	0	2
BMI assessment		
Normal	18 (22.5%)	15 (19.2%)
Overweight	26 (32.5%)	32 (41.0%)
Obesity	36 (45.0%)	31 (39.8%)
Missing data	3	5

**Table 3:** Prevalence of obesity-related complications.

	PAQ-Ch	PAQ-A	Whole Group (PAQ-Ch and PAQ-A)
Hypertension			
Yes	1 (2.4%)	13 (32.5%)	14 (17.3%)
No	40 (97.6%)	27 (67.5%)	67 (82.7%)
Missing data	1	1	2
Hypercholesterolemia			
Yes	3 (7.5%)	3 (7.3%)	6 (7.4%)
No	37 (92.5%)	38 (92.7%)	75 (92.6%)
Missing data	2	0	2
Hypothyroidism			
Yes	3 (7.5%)	5 (12.5%)	8 (10%)
No	37 (92.5%)	35 (87.5%)	72 (90%)
Missing data	2	1	3
Hyperinsulinemia			
Yes	2 (5%)	6 (14.6%)	8 (9.9%)
No	38 (95%)	35 (85.4%)	73 (90.1%)
Missing data	2	0	2

**Table 4:** Parental physical activity level.

	Mother	Father
IPAQ physical activity level		
High	12 (14.6%)	23 (37.7%)
Moderate	41 (50.00%)	20 (32.8%)
Low	29 (35.4%)	18 (29.5%)
Missing data	1	22

**Table 5:** Patients’ physical activity level according to PAQ-Ch or PAQ-A.

	PAQ-Ch and PAQ-A	PAQ-Ch	PAQ-A
Mean ± SD	2.09 ± 0.69	2.23 ± 0.66	1.96 ± 0.71
Median [Q1; Q3]	1.91 [1.66; 2.50]	2.31 [1.79; 2.60]	1.85 [1.56; 2.27]
Min–Max	1.00–4.15	1.00–4.15	1.00–3.92

**Table 6:** Spearman’s correlation coefficients (rho) examining the bivariate associations between the final PAQ-Ch or PAQ-A score and selected parental and environmental parameters.

	PAQ-Ch		PAQ-A	
	rho	*p*	rho	*p*
Mother’s education	−0.28	0.08	0.31	<0.05
Mother’s physical activity	0.05	0.77	0.21	0.20
Mother’s BMI assessment	−0.25	0.12	0.18	0.28
Father’s education	0.08	0.63	0.05	0.75
Father’s physical activity	0.38	<0.05	−0.06	0.75
Father’s BMI assessment	−0.04	0.79	0.08	0.64
Place of residence	0.20	0.20	0.10	0.52
Distance from home to school	0.17	0.29	0.07	0.67

**Table 7:** Final regression model to predict patient’s PAQ.

Variable in the Final Model	β	Std. Err. β	*p*	−95% CI	+95% CI	Final Model
Patient’s age	−0.24	0.11	0.04	−0.47	−0.01	R^2^ = 0.04 F = 4.26 *p* = 0.04

β—regression coefficient; Std. Err.—standard error; CI—confidence interval.

## Data Availability

The data presented in this study are available upon request from the corresponding author.

## Supplementary Materials

The following supporting information can be downloaded at: https://www.mdpi.com/article/10.3390/healthcare12020260/s1, Table S1. A stepwise backward method in building the final regression model—steps from 1 to 10.
