# Supplementary material for: Physical Activity and Its Potential Determinants in Obese Children and Adolescents under Specialist Outpatient Care—A Pilot Cross-Sectional Study
Source: Healthcare (Basel). 2024 Jan 19;12(2):260. doi: 10.3390/healthcare12020260 (PMC10815763; doi:10.3390/healthcare12020260)
Supplement: Supplementary file 1 [file healthcare-12-00260-s001.zip › healthcare-2757058-supplementary.pdf]

Table S1. A stepwise backward method in building the final regression model – steps from 1 to 10.

Variable	F to remove	p-value to remove	effect
**STEP 1**			
Gender	0.539	0.466	in model
Age	3.081	0.084	in model
At least one parent with higher education	0.405	0.527	in model
Mother's working status	1.438	0.235	in model
Father's working status	0.777	0.381	in model
At least one obese parent	0.522	0.473	in model
Place of residence	1.056	0.374	in model
Distance from home to school	0.008	0.992	to remove
Mode of commuting to school	0.022	0.882	in model
At least one parent with moderate or high physical activity level	0.234	0.630	in model
**STEP 2**			
Gender	0.545	0.463	in model
Age	3.501	0.066	in model
At least one parent with higher education	0.424	0.517	in model
Mother's working status	1.610	0.209	in model
Father's working status	0.789	0.378	in model
At least one obese parent	0.557	0.458	in model
Place of residence	1.100	0.356	in model
Distance from home to school			out of model
Mode of commuting to school	0.141	0.708	to remove

At least one parent with moderate or high physical activity level	0.232	0.632	in model
**STEP 3**			
Gender	0.676	0.414	in model
Age	3.588	0.063	in model
At least one parent with higher education	0.424	0.517	in model
Mother's working status	1.743	0.192	in model
Father's working status	0.825	0.367	in model
At least one obese parent	0.527	0.471	in model
Place of residence	1.260	0.296	in model
Distance from home to school			out of model
Mode of commuting to school			out of model
At least one parent with moderate or high physical activity level	0.203	0.654	to remove
**STEP 4**			
Gender	0.645	0.425	in model
Age	4.618	0.035	in model
At least one parent with higher education	0.506	0.474	to remove
Mother's working status	1.881	0.175	in model
Father's working status	0.795	0.376	in model
At least one obese parent	0.652	0.422	in model
Place of residence	1.320	0.276	in model
Distance from home to school			out of model
Mode of commuting to school			out of model
At least one parent with moderate or high physical activity level			out of model
**STEP 5**			

Gender	0.983	0.325	in model
Age	4.696	0.034	in model
At least one parent with higher education			out of model
Mother's working status	2.675	0.107	in model
Father's working status	1.035	0.313	in model
At least one obese parent	0.546	0.463	to remove
Place of residence	1.286	0.287	in model
Distance from home to school			out of model
Mode of commuting to school			out of model
At least one parent with moderate or high physical activity level			out of model
**STEP 6**			
Gender	1.064	0.306	in model
Age	4.420	0.039	in model
At least one parent with higher education			out of model
Mother's working status	2.657	0.108	in model
Father's working status	0.884	0.350	to remove
At least one obese parent			out of model
Place of residence	1.318	0.276	in model
Distance from home to school			out of model
Mode of commuting to school			out of model
At least one parent with moderate or high physical activity level			out of model
**STEP 7**			
Gender	1.120	0.294	to remove
Age	4.822	0.032	in model
At least one parent with higher education			out of model

Mother's working status	2.424	0.124	in model
Father's working status			out of model
At least one obese parent			out of model
Place of residence	1.322	0.274	in model
Distance from home to school			out of model
Mode of commuting to school			out of model
At least one parent with moderate or high physical activity level			out of model
**STEP 8**			
Gender			out of model
Age	4.661	0.034	in model
At least one parent with higher education			out of model
Mother's working status	2.391	0.127	in model
Father's working status			out of model
At least one obese parent			out of model
Place of residence	1.065	0.370	to remove
Distance from home to school			out of model
Mode of commuting to school			out of model
At least one parent with moderate or high physical activity level			out of model
**STEP 9**			
Gender			out of model
Age	4.534	0.037	in model
At least one parent with higher education			out of model
Mother's working status	3.235	0.076	to remove
Father's working status			out of model
At least one obese parent			out of model
Place of residence			out of model

Distance from home to school			out of model
Mode of commuting to school			out of model
At least one parent with moderate or high physical activity level			out of model
**STEP 10**			
Gender			out of model
Age	4.264	0.043	in model
At least one parent with higher education			out of model
Mother's working status			out of model
Father's working status			out of model
At least one obese parent			out of model
Place of residence			out of model
Distance from home to school			out of model
Mode of commuting to school			out of model
At least one parent with moderate or high physical activity level			out of model
